# A model of propagating waves in cerebral cortex across network states

**DOI:** 10.1186/1471-2202-12-S1-P67

**Published:** 2011-07-18

**Authors:** Lyle E Muller, Alain Destexhe

**Affiliations:** 1 UNIC, CNRS, Gif-sur-Yvette, 91198, France

## 

Propagating waves of activity have been measured in cerebral cortex in various experimental preparations, and show different propagation speed or patterns. Using network models, we investigated whether the “state” of the network can explain these differences. We used a modified version of a previous model, in which neuronal adaptation can facilitate the transition from activated, asynchronous irregular (AI) states to quiescence in random networks. [1] With a proper re-ignition mechanism, these networks can transition between UP/DOWN and AI states with different levels of adaptation. Here, we have studied the occurrence of propagating waves during this transition from UP/DOWN to self-sustained activated states in topographic spiking neural network models and compared these results to voltage-sensitive dye imaging data from the visual cortex, as well as to other known experimental results. The addition of local connections with realistic synaptic delays in these topographic spiking neural network models allows for the possibility of propagating slow waves in some network states. Further, while it is generally thought that the large, low-frequency propagation evoked by sensory stimulation during heavily anesthetized states gives way to a bump attractor during waking, activated states, we demonstrate the possibility that propagating activity exists throughout the whole spectrum of network activation and shifts from low frequency (predominantly controlled by adaptation) to high frequency (predominantly controlled by E/I interactions) as the level of network activation increases. With these results from network modeling, we aim to account both for observed effects of anesthesia on the spread of cortical activity in voltage-sensitive dye and electrophysiological experiments [2-4] and for the observation of traveling high-frequency oscillations in vitro [5] and in awake monkeys [6,7].
